# Supplementary material for: Comparative Proteomics Study of Yak Milk from Standard and Naturally Extended Lactation Using iTRAQ Technique
Source: Animals (Basel). 2022 Feb 7;12(3):391. doi: 10.3390/ani12030391 (PMC8833776; doi:10.3390/ani12030391)
Supplement: Supplementary file 1 [file animals-12-00391-s001.zip › Supplemental material 7-KEGG Enrichment.htm]

B-VS-Q

1. B-VS-Q

| # | Pathway | Sample1 (200) | Sample2 (735) | Pvalue | Pathway ID |
| 1 | Carbon metabolism | 21 | 38 | 0.0001628096 | ko01200 |
| 2 | Biosynthesis of amino acids | 14 | 23 | 0.0005961622 | ko01230 |
| 3 | Hypertrophic cardiomyopathy (HCM) | 19 | 37 | 0.001179062 | ko05410 |
| 4 | Dilated cardiomyopathy | 21 | 45 | 0.003087347 | ko05414 |
| 5 | Thyroid hormone signaling pathway | 12 | 22 | 0.005420035 | ko04919 |
| 6 | Glycine, serine and threonine metabolism | 5 | 6 | 0.00670732 | ko00260 |
| 7 | Cardiac muscle contraction | 7 | 11 | 0.01181832 | ko04260 |
| 8 | Arrhythmogenic right ventricular cardiomyopathy (ARVC) | 18 | 41 | 0.01338392 | ko05412 |
| 9 | Amyotrophic lateral sclerosis (ALS) | 6 | 9 | 0.01490736 | ko05014 |
| 10 | Focal adhesion | 21 | 51 | 0.01786332 | ko04510 |
| 11 | 2-Oxocarboxylic acid metabolism | 5 | 7 | 0.01828684 | ko01210 |
| 12 | Arginine biosynthesis | 3 | 3 | 0.01992787 | ko00220 |
| 13 | Tyrosine metabolism | 3 | 3 | 0.01992787 | ko00350 |
| 14 | Phenylalanine metabolism | 3 | 3 | 0.01992787 | ko00360 |
| 15 | Legionellosis | 9 | 17 | 0.02042987 | ko05134 |
| 16 | Mineral absorption | 4 | 5 | 0.02106356 | ko04978 |
| 17 | Fat digestion and absorption | 4 | 5 | 0.02106356 | ko04975 |
| 18 | Glucagon signaling pathway | 7 | 12 | 0.0218907 | ko04922 |
| 19 | Osteoclast differentiation | 8 | 15 | 0.02732169 | ko04380 |
| 20 | Glycolysis / Gluconeogenesis | 10 | 21 | 0.03433107 | ko00010 |
| 21 | Regulation of actin cytoskeleton | 22 | 57 | 0.03458404 | ko04810 |
| 22 | Central carbon metabolism in cancer | 7 | 13 | 0.03665795 | ko05230 |
| 23 | Citrate cycle (TCA cycle) | 7 | 13 | 0.03665795 | ko00020 |
| 24 | Malaria | 8 | 16 | 0.04211339 | ko05144 |
| 25 | HIF-1 signaling pathway | 8 | 16 | 0.04211339 | ko04066 |
| 26 | Leukocyte transendothelial migration | 17 | 44 | 0.06009835 | ko04670 |
| 27 | p53 signaling pathway | 3 | 4 | 0.06362218 | ko04115 |
| 28 | Alanine, aspartate and glutamate metabolism | 3 | 4 | 0.06362218 | ko00250 |
| 29 | Metabolic pathways | 40 | 120 | 0.06402548 | ko01100 |
| 30 | Complement and coagulation cascades | 13 | 32 | 0.06539955 | ko04610 |
| 31 | Bile secretion | 2 | 2 | 0.07377338 | ko04976 |
| 32 | Phenylalanine, tyrosine and tryptophan biosynthesis | 2 | 2 | 0.07377338 | ko00400 |
| 33 | Pathogenic Escherichia coli infection | 16 | 42 | 0.07597506 | ko05130 |
| 34 | Protein processing in endoplasmic reticulum | 13 | 33 | 0.0825477 | ko04141 |
| 35 | Proteoglycans in cancer | 18 | 50 | 0.1018915 | ko05205 |
| 36 | ECM-receptor interaction | 13 | 34 | 0.1023166 | ko04512 |
| 37 | Toxoplasmosis | 12 | 31 | 0.1054765 | ko05145 |
| 38 | MAPK signaling pathway | 9 | 22 | 0.1130094 | ko04010 |
| 39 | Influenza A | 7 | 16 | 0.1139174 | ko05164 |
| 40 | Pertussis | 14 | 38 | 0.1199563 | ko05133 |
| 41 | Thyroid hormone synthesis | 3 | 5 | 0.1274601 | ko04918 |
| 42 | Small cell lung cancer | 9 | 23 | 0.1434665 | ko05222 |
| 43 | Non-alcoholic fatty liver disease (NAFLD) | 8 | 20 | 0.1474802 | ko04932 |
| 44 | Bacterial invasion of epithelial cells | 12 | 33 | 0.1563655 | ko05100 |
| 45 | Vitamin digestion and absorption | 2 | 3 | 0.1814644 | ko04977 |
| 46 | Neuroactive ligand-receptor interaction | 2 | 3 | 0.1814644 | ko04080 |
| 47 | Proximal tubule bicarbonate reclamation | 2 | 3 | 0.1814644 | ko04964 |
| 48 | Adrenergic signaling in cardiomyocytes | 6 | 15 | 0.1990922 | ko04261 |
| 49 | Glyoxylate and dicarboxylate metabolism | 3 | 6 | 0.2051441 | ko00630 |
| 50 | Salivary secretion | 3 | 6 | 0.2051441 | ko04970 |
| 51 | Leishmaniasis | 13 | 38 | 0.2068912 | ko05140 |
| 52 | Phagosome | 20 | 62 | 0.2144009 | ko04145 |
| 53 | MicroRNAs in cancer | 8 | 22 | 0.2261131 | ko05206 |
| 54 | PI3K-Akt signaling pathway | 16 | 49 | 0.2326416 | ko04151 |
| 55 | Salmonella infection | 7 | 19 | 0.2377916 | ko05132 |
| 56 | Alzheimer's disease | 10 | 29 | 0.2418827 | ko05010 |
| 57 | cAMP signaling pathway | 6 | 16 | 0.2501971 | ko04024 |
| 58 | Shigellosis | 11 | 33 | 0.2658673 | ko05131 |
| 59 | Fatty acid biosynthesis | 1 | 1 | 0.2721088 | ko00061 |
| 60 | Protein export | 1 | 1 | 0.2721088 | ko03060 |
| 61 | Nicotine addiction | 1 | 1 | 0.2721088 | ko05033 |
| 62 | D-Glutamine and D-glutamate metabolism | 1 | 1 | 0.2721088 | ko00471 |
| 63 | ABC transporters | 1 | 1 | 0.2721088 | ko02010 |
| 64 | Nitrogen metabolism | 1 | 1 | 0.2721088 | ko00910 |
| 65 | Pyruvate metabolism | 4 | 10 | 0.2769142 | ko00620 |
| 66 | Viral myocarditis | 10 | 30 | 0.2811926 | ko05416 |
| 67 | Platelet activation | 12 | 37 | 0.2875713 | ko04611 |
| 68 | Peroxisome | 3 | 7 | 0.290181 | ko04146 |
| 69 | Huntington's disease | 9 | 27 | 0.2979588 | ko05016 |
| 70 | Fatty acid metabolism | 2 | 4 | 0.2993066 | ko01212 |
| 71 | cGMP-PKG signaling pathway | 6 | 17 | 0.3044746 | ko04022 |
| 72 | Herpes simplex infection | 5 | 14 | 0.3248888 | ko05168 |
| 73 | Hepatitis B | 4 | 11 | 0.3485366 | ko05161 |
| 74 | Cysteine and methionine metabolism | 4 | 11 | 0.3485366 | ko00270 |
| 75 | Protein digestion and absorption | 4 | 11 | 0.3485366 | ko04974 |
| 76 | Antigen processing and presentation | 6 | 18 | 0.3605916 | ko04612 |
| 77 | Propanoate metabolism | 3 | 8 | 0.3770167 | ko00640 |
| 78 | Insulin resistance | 3 | 8 | 0.3770167 | ko04931 |
| 79 | Fructose and mannose metabolism | 3 | 8 | 0.3770167 | ko00051 |
| 80 | AMPK signaling pathway | 5 | 15 | 0.3877871 | ko04152 |
| 81 | Parkinson's disease | 7 | 22 | 0.3889511 | ko05012 |
| 82 | Rap1 signaling pathway | 15 | 51 | 0.4116406 | ko04015 |
| 83 | Staphylococcus aureus infection | 8 | 26 | 0.4124788 | ko05150 |
| 84 | Endocrine and other factor-regulated calcium reabsorption | 2 | 5 | 0.413871 | ko04961 |
| 85 | NOD-like receptor signaling pathway | 2 | 5 | 0.413871 | ko04621 |
| 86 | Tryptophan metabolism | 2 | 5 | 0.413871 | ko00380 |
| 87 | Prolactin signaling pathway | 2 | 5 | 0.413871 | ko04917 |
| 88 | Oxytocin signaling pathway | 4 | 12 | 0.4203569 | ko04921 |
| 89 | Measles | 4 | 12 | 0.4203569 | ko05162 |
| 90 | Estrogen signaling pathway | 4 | 12 | 0.4203569 | ko04915 |
| 91 | Spliceosome | 7 | 23 | 0.4409086 | ko03040 |
| 92 | Tight junction | 7 | 23 | 0.4409086 | ko04530 |
| 93 | Cell adhesion molecules (CAMs) | 13 | 45 | 0.4557489 | ko04514 |
| 94 | Hematopoietic cell lineage | 8 | 27 | 0.4606978 | ko04640 |
| 95 | Gap junction | 3 | 9 | 0.4614237 | ko04540 |
| 96 | Glutathione metabolism | 3 | 9 | 0.4614237 | ko00480 |
| 97 | Arginine and proline metabolism | 3 | 9 | 0.4614237 | ko00330 |
| 98 | Inositol phosphate metabolism | 1 | 2 | 0.4704443 | ko00562 |
| 99 | Carbohydrate digestion and absorption | 1 | 2 | 0.4704443 | ko04973 |
| 100 | Fatty acid elongation | 1 | 2 | 0.4704443 | ko00062 |
| 101 | Inflammatory bowel disease (IBD) | 1 | 2 | 0.4704443 | ko05321 |
| 102 | Vascular smooth muscle contraction | 4 | 13 | 0.4901573 | ko04270 |
| 103 | Pathways in cancer | 13 | 46 | 0.4931287 | ko05200 |
| 104 | Calcium signaling pathway | 5 | 17 | 0.5111003 | ko04020 |
| 105 | Oxidative phosphorylation | 5 | 17 | 0.5111003 | ko00190 |
| 106 | Hippo signaling pathway | 5 | 17 | 0.5111003 | ko04390 |
| 107 | Gastric acid secretion | 2 | 6 | 0.5182344 | ko04971 |
| 108 | Hippo signaling pathway - fly | 2 | 6 | 0.5182344 | ko04391 |
| 109 | Starch and sucrose metabolism | 2 | 6 | 0.5182344 | ko00500 |
| 110 | Pancreatic secretion | 2 | 6 | 0.5182344 | ko04972 |
| 111 | TNF signaling pathway | 3 | 10 | 0.5404992 | ko04668 |
| 112 | Viral carcinogenesis | 8 | 29 | 0.5542184 | ko05203 |
| 113 | PPAR signaling pathway | 4 | 14 | 0.5562647 | ko03320 |
| 114 | Neurotrophin signaling pathway | 4 | 14 | 0.5562647 | ko04722 |
| 115 | Axon guidance | 9 | 34 | 0.606588 | ko04360 |
| 116 | Toll-like receptor signaling pathway | 2 | 7 | 0.6094557 | ko04620 |
| 117 | Valine, leucine and isoleucine degradation | 2 | 7 | 0.6094557 | ko00280 |
| 118 | Chemokine signaling pathway | 3 | 11 | 0.6124852 | ko04062 |
| 119 | Prostate cancer | 3 | 11 | 0.6124852 | ko05215 |
| 120 | Glycosaminoglycan degradation | 1 | 3 | 0.6149343 | ko00531 |
| 121 | Cocaine addiction | 1 | 3 | 0.6149343 | ko05030 |
| 122 | Sulfur metabolism | 1 | 3 | 0.6149343 | ko00920 |
| 123 | Drug metabolism - cytochrome P450 | 1 | 3 | 0.6149343 | ko00982 |
| 124 | Ether lipid metabolism | 1 | 3 | 0.6149343 | ko00565 |
| 125 | Adipocytokine signaling pathway | 1 | 3 | 0.6149343 | ko04920 |
| 126 | Phototransduction - fly | 1 | 3 | 0.6149343 | ko04745 |
| 127 | Renin-angiotensin system | 1 | 3 | 0.6149343 | ko04614 |
| 128 | Insulin signaling pathway | 4 | 15 | 0.6175293 | ko04910 |
| 129 | Natural killer cell mediated cytotoxicity | 6 | 23 | 0.6289668 | ko04650 |
| 130 | B cell receptor signaling pathway | 3 | 12 | 0.676528 | ko04662 |
| 131 | Purine metabolism | 2 | 8 | 0.6869354 | ko00230 |
| 132 | Pentose phosphate pathway | 2 | 8 | 0.6869354 | ko00030 |
| 133 | Adherens junction | 6 | 25 | 0.7164616 | ko04520 |
| 134 | Amoebiasis | 8 | 33 | 0.7165216 | ko05146 |
| 135 | N-Glycan biosynthesis | 1 | 4 | 0.7201435 | ko00510 |
| 136 | Chemical carcinogenesis | 1 | 4 | 0.7201435 | ko05204 |
| 137 | Metabolism of xenobiotics by cytochrome P450 | 1 | 4 | 0.7201435 | ko00980 |
| 138 | Vibrio cholerae infection | 1 | 4 | 0.7201435 | ko05110 |
| 139 | Aldosterone-regulated sodium reabsorption | 1 | 4 | 0.7201435 | ko04960 |
| 140 | Lysine degradation | 1 | 4 | 0.7201435 | ko00310 |
| 141 | Amphetamine addiction | 1 | 4 | 0.7201435 | ko05031 |
| 142 | Fatty acid degradation | 1 | 4 | 0.7201435 | ko00071 |
| 143 | Jak-STAT signaling pathway | 1 | 4 | 0.7201435 | ko04630 |
| 144 | Bladder cancer | 1 | 4 | 0.7201435 | ko05219 |
| 145 | Pyrimidine metabolism | 1 | 4 | 0.7201435 | ko00240 |
| 146 | Cell cycle | 1 | 4 | 0.7201435 | ko04110 |
| 147 | African trypanosomiasis | 3 | 13 | 0.7324392 | ko05143 |
| 148 | Galactose metabolism | 2 | 9 | 0.7513673 | ko00052 |
| 149 | Primary immunodeficiency | 4 | 18 | 0.7672081 | ko05340 |
| 150 | Rheumatoid arthritis | 4 | 18 | 0.7672081 | ko05323 |
| 151 | Sphingolipid signaling pathway | 3 | 14 | 0.7804868 | ko04071 |
| 152 | Hepatitis C | 1 | 5 | 0.7967116 | ko05160 |
| 153 | Synaptic vesicle cycle | 1 | 5 | 0.7967116 | ko04721 |
| 154 | Insulin secretion | 1 | 5 | 0.7967116 | ko04911 |
| 155 | Progesterone-mediated oocyte maturation | 1 | 5 | 0.7967116 | ko04914 |
| 156 | Apoptosis | 1 | 5 | 0.7967116 | ko04210 |
| 157 | T cell receptor signaling pathway | 2 | 10 | 0.8040843 | ko04660 |
| 158 | Systemic lupus erythematosus | 6 | 28 | 0.8192305 | ko05322 |
| 159 | Alcoholism | 3 | 15 | 0.8212262 | ko05034 |
| 160 | HTLV-I infection | 5 | 24 | 0.8273062 | ko05166 |
| 161 | Tuberculosis | 7 | 33 | 0.839556 | ko05152 |
| 162 | Intestinal immune network for IgA production | 2 | 11 | 0.8466619 | ko04672 |
| 163 | mRNA surveillance pathway | 1 | 6 | 0.852407 | ko03015 |
| 164 | FoxO signaling pathway | 1 | 6 | 0.852407 | ko04068 |
| 165 | Graft-versus-host disease | 1 | 6 | 0.852407 | ko05332 |
| 166 | Type II diabetes mellitus | 1 | 6 | 0.852407 | ko04930 |
| 167 | Type I diabetes mellitus | 1 | 6 | 0.852407 | ko04940 |
| 168 | Glutamatergic synapse | 1 | 6 | 0.852407 | ko04724 |
| 169 | Long-term potentiation | 1 | 6 | 0.852407 | ko04720 |
| 170 | TGF-beta signaling pathway | 1 | 6 | 0.852407 | ko04350 |
| 171 | Pancreatic cancer | 1 | 6 | 0.852407 | ko05212 |
| 172 | Ribosome | 3 | 16 | 0.8553716 | ko03010 |
| 173 | Epstein-Barr virus infection | 6 | 30 | 0.8697683 | ko05169 |
| 174 | Autoimmune thyroid disease | 2 | 12 | 0.8806887 | ko05320 |
| 175 | Allograft rejection | 2 | 12 | 0.8806887 | ko05330 |
| 176 | Transcriptional misregulation in cancer | 3 | 17 | 0.8837028 | ko05202 |
| 177 | beta-Alanine metabolism | 1 | 7 | 0.892899 | ko00410 |
| 178 | Choline metabolism in cancer | 1 | 7 | 0.892899 | ko05231 |
| 179 | Arachidonic acid metabolism | 1 | 7 | 0.892899 | ko00590 |
| 180 | RNA degradation | 1 | 7 | 0.892899 | ko03018 |
| 181 | Prion diseases | 3 | 18 | 0.9070017 | ko05020 |
| 182 | Fc epsilon RI signaling pathway | 2 | 13 | 0.9076431 | ko04664 |
| 183 | Circadian entrainment | 1 | 8 | 0.9223223 | ko04713 |
| 184 | Chagas disease (American trypanosomiasis) | 1 | 8 | 0.9223223 | ko05142 |
| 185 | Colorectal cancer | 1 | 8 | 0.9223223 | ko05210 |
| 186 | Vasopressin-regulated water reabsorption | 1 | 8 | 0.9223223 | ko04962 |
| 187 | Oocyte meiosis | 1 | 8 | 0.9223223 | ko04114 |
| 188 | Endocytosis | 6 | 33 | 0.9232467 | ko04144 |
| 189 | Fc gamma R-mediated phagocytosis | 3 | 19 | 0.9260113 | ko04666 |
| 190 | NF-kappa B signaling pathway | 1 | 9 | 0.9436917 | ko04064 |
| 191 | Asthma | 1 | 9 | 0.9436917 | ko05310 |
| 192 | VEGF signaling pathway | 1 | 9 | 0.9436917 | ko04370 |
| 193 | RNA transport | 2 | 15 | 0.9453911 | ko03013 |
| 194 | Proteasome | 1 | 10 | 0.9592036 | ko03050 |
| 195 | Lysosome | 3 | 23 | 0.971628 | ko04142 |
| 196 | Phospholipase D signaling pathway | 1 | 15 | 0.9919196 | ko04072 |

| # | Pathway | Proteins |
| --- | --- | --- |
| 1 | Carbon metabolism (no map in kegg database) | ENSBTAP00000017392-D1, ENSP00000414662-D1, ENSBTAP00000033696-D1, ENSBTAP00000052439-D3, ENSBTAP00000027941-D1, ENSP00000331897-D1, ENSBTAP00000009923-D1, ENSBTAP00000007982-D1, ENSBTAP00000004431-D1, ENSBTAP00000037577-D6, ENSP00000345771-D1, ENSBTAP00000005730-D1, ENSP00000403265-D1, ENSP00000359991-D1, ENSP00000362413-D2, ENSBTAP00000009440-D1, ENSBTAP00000015873-D1, ENSBTAP00000008907-D1, ENSBTAP00000027348-D1, ENSP00000229270-D1, ENSP00000378669-D1 |
| 2 | Biosynthesis of amino acids (no map in kegg database) | ENSBTAP00000017392-D1, ENSP00000331897-D1, ENSBTAP00000037577-D6, ENSP00000345771-D1, ENSBTAP00000005730-D1, ENSP00000403265-D1, ENSP00000359991-D1, ENSP00000362413-D2, ENSBTAP00000009440-D1, ENSBTAP00000015873-D1, ENSBTAP00000008907-D1, ENSBTAP00000027348-D1, ENSP00000229270-D1, ENSP00000378669-D1 |
| 3 | Hypertrophic cardiomyopathy (HCM) | ENSP00000343009-D1, ENSBTAP00000019704-D1, ENSBTAP00000015668-D1, ENSP00000355330-D1, ENSBTAP00000053653-D7, ENSP00000346839-D1, ENSBTAP00000006923-D1, ENSP00000260356-D1, ENSP00000350667-D1, ENSP00000350667-D3, ENSP00000363827-D1, ENSBTAP00000008132-D2, ENSP00000300527-D1, ENSP00000265132-D1, ENSBTAP00000009406-D1, ENSBTAP00000050318-D1, ENSP00000306099-D1, ENSBTAP00000023373-D1, ENSP00000306361-D1 |
| 4 | Dilated cardiomyopathy | ENSP00000343009-D1, ENSBTAP00000019704-D1, ENSBTAP00000015668-D1, ENSP00000355330-D1, ENSBTAP00000053653-D7, ENSP00000346839-D1, ENSBTAP00000006923-D1, ENSP00000260356-D1, ENSP00000350667-D1, ENSP00000350667-D3, ENSP00000363827-D1, ENSBTAP00000008132-D2, ENSBTAP00000030521-D1, ENSP00000300527-D1, ENSP00000265132-D1, ENSBTAP00000009406-D1, ENSBTAP00000050318-D1, ENSBTAP00000017182-D1, ENSP00000306099-D1, ENSBTAP00000023373-D1, ENSP00000306361-D1 |
| 5 | Thyroid hormone signaling pathway | ENSBTAP00000019704-D1, ENSBTAP00000053653-D7, ENSP00000346839-D1, ENSBTAP00000006923-D1, ENSBTAP00000013169-D1, ENSP00000345771-D1, ENSBTAP00000008132-D2, ENSBTAP00000009406-D1, ENSBTAP00000017182-D1, ENSP00000306099-D1, ENSBTAP00000010351-D1, ENSP00000306361-D1 |
| 6 | Glycine, serine and threonine metabolism | ENSBTAP00000017392-D1, ENSBTAP00000033696-D1, ENSBTAP00000001383-D1, ENSP00000359991-D1, ENSBTAP00000008907-D1 |
| 7 | Cardiac muscle contraction | ENSP00000317159-D1, ENSBTAP00000019704-D1, ENSP00000306397-D1, ENSBTAP00000053653-D7, ENSBTAP00000013169-D1, ENSP00000350667-D1, ENSP00000350667-D3 |
| 8 | Arrhythmogenic right ventricular cardiomyopathy (ARVC) | ENSP00000343009-D1, ENSBTAP00000019704-D1, ENSP00000369129-D1, ENSBTAP00000015668-D1, ENSP00000355330-D1, ENSP00000346839-D1, ENSBTAP00000006923-D1, ENSP00000260356-D1, ENSP00000363827-D1, ENSBTAP00000008132-D2, ENSP00000300527-D1, ENSP00000265132-D1, ENSBTAP00000009406-D1, ENSBTAP00000050318-D1, ENSBTAP00000049867-D3, ENSP00000306099-D1, ENSBTAP00000023373-D1, ENSP00000306361-D1 |
| 9 | Amyotrophic lateral sclerosis (ALS) | ENSP00000405382-D2, ENSP00000293190-D1, ENSBTAP00000027941-D1, ENSBTAP00000014883-D1, ENSP00000357076-D4, yakG038520 |
| 10 | Focal adhesion | ENSP00000343009-D1, ENSBTAP00000024902-D1, ENSBTAP00000015668-D1, ENSP00000211998-D1, ENSP00000420213-D1, ENSP00000357076-D4, ENSP00000355330-D1, ENSP00000346839-D1, ENSBTAP00000006923-D1, ENSP00000260356-D1, ENSP00000363827-D1, ENSBTAP00000008132-D2, ENSP00000300527-D1, ENSP00000265132-D1, ENSBTAP00000009406-D1, ENSBTAP00000015248-D2, ENSBTAP00000049867-D3, ENSP00000306099-D1, ENSP00000358879-D1, ENSP00000327145-D1, ENSP00000306361-D1 |
| 11 | 2-Oxocarboxylic acid metabolism (no map in kegg database) | ENSP00000331897-D1, ENSBTAP00000005730-D1, ENSBTAP00000009440-D1, ENSBTAP00000015873-D1, ENSBTAP00000027348-D1 |
| 12 | Arginine biosynthesis | ENSBTAP00000009923-D1, ENSBTAP00000009440-D1, ENSBTAP00000015873-D1 |
| 13 | Tyrosine metabolism | ENSBTAP00000001383-D1, ENSBTAP00000009440-D1, ENSBTAP00000015873-D1 |
| 14 | Phenylalanine metabolism | ENSBTAP00000001383-D1, ENSBTAP00000009440-D1, ENSBTAP00000015873-D1 |
| 15 | Legionellosis | ENSP00000405382-D2, ENSP00000310226-D1, ENSBTAP00000017497-D1, ENSP00000364805-D5, ENSBTAP00000022979-D1, ENSBTAP00000019970-D1, ENSP00000339053-D13, ENSP00000306099-D1, ENSP00000306361-D1 |
| 16 | Mineral absorption | ENSBTAP00000013169-D1, ENSBTAP00000009564-D1, ENSBTAP00000001704-D2, ENSBTAP00000002023-D1 |
| 17 | Fat digestion and absorption | ENSBTAP00000026345-D1, ENSBTAP00000023750-D1, ENSBTAP00000009440-D1, ENSBTAP00000038799-D1 |
| 18 | Glucagon signaling pathway | ENSBTAP00000052439-D3, ENSBTAP00000015277-D1, ENSP00000216962-D1, ENSP00000395337-D3, ENSP00000345771-D1, ENSP00000403265-D1, ENSP00000359991-D1 |
| 19 | Osteoclast differentiation | ENSBTAP00000001440-D1, ENSP00000346839-D1, ENSBTAP00000006923-D1, ENSBTAP00000012837-D1, ENSBTAP00000009406-D1, ENSP00000306099-D1, ENSBTAP00000010351-D1, ENSP00000306361-D1 |
| 20 | Glycolysis / Gluconeogenesis | ENSBTAP00000033696-D1, ENSBTAP00000052439-D3, ENSP00000395337-D3, ENSBTAP00000037577-D6, ENSP00000345771-D1, ENSP00000403265-D1, ENSP00000359991-D1, ENSP00000362413-D2, ENSP00000229270-D1, ENSP00000378669-D1 |
| 21 | Regulation of actin cytoskeleton | ENSP00000343009-D1, ENSBTAP00000024902-D1, ENSBTAP00000015668-D1, ENSP00000211998-D1, ENSP00000357076-D4, ENSP00000355330-D1, ENSP00000346839-D1, ENSBTAP00000006923-D1, ENSP00000260356-D1, ENSBTAP00000022979-D1, ENSBTAP00000020512-D4, ENSP00000363827-D1, ENSBTAP00000008132-D2, ENSP00000300527-D1, ENSP00000265132-D1, ENSBTAP00000009406-D1, ENSBTAP00000015248-D2, ENSBTAP00000049867-D3, ENSP00000306099-D1, ENSBTAP00000006465-D4, ENSP00000379613-D3, ENSP00000306361-D1 |
| 22 | Central carbon metabolism in cancer | ENSBTAP00000052439-D3, ENSP00000331897-D1, ENSP00000395337-D3, ENSP00000345771-D1, ENSP00000403265-D1, ENSP00000359991-D1, ENSBTAP00000027348-D1 |
| 23 | Citrate cycle (TCA cycle) | ENSP00000414662-D1, ENSBTAP00000033696-D1, ENSBTAP00000052439-D3, ENSP00000331897-D1, ENSBTAP00000007982-D1, ENSBTAP00000005730-D1, ENSBTAP00000027348-D1 |
| 24 | Malaria | ENSBTAP00000050256-D2, ENSBTAP00000051337-D10, ENSP00000346839-D1, ENSP00000260356-D1, ENSBTAP00000022979-D1, ENSBTAP00000023750-D1, ENSP00000306099-D1, ENSP00000306361-D1 |
| 25 | HIF-1 signaling pathway | ENSBTAP00000052439-D3, ENSP00000395337-D3, ENSBTAP00000037577-D6, ENSP00000345771-D1, ENSBTAP00000009564-D1, ENSP00000362413-D2, ENSBTAP00000001704-D2, ENSP00000378669-D1 |
| 26 | Leukocyte transendothelial migration | ENSBTAP00000024902-D1, ENSBTAP00000015668-D1, ENSP00000211998-D1, ENSP00000357076-D4, ENSP00000355330-D1, ENSP00000346839-D1, ENSBTAP00000006923-D1, ENSP00000260356-D1, ENSBTAP00000022979-D1, ENSP00000363827-D1, ENSBTAP00000008132-D2, ENSP00000300527-D1, ENSP00000265132-D1, ENSBTAP00000015248-D2, ENSBTAP00000049867-D3, ENSP00000306099-D1, ENSP00000306361-D1 |
| 27 | p53 signaling pathway | ENSP00000405382-D2, ENSP00000260356-D1, ENSBTAP00000009715-D1 |
| 28 | Alanine, aspartate and glutamate metabolism | ENSBTAP00000009923-D1, ENSBTAP00000009440-D1, ENSBTAP00000015873-D1 |
| 29 | Metabolic pathways (no map in kegg database) | ENSP00000317159-D1, ENSP00000405382-D2, ENSBTAP00000017392-D1, ENSP00000414662-D1, ENSBTAP00000033696-D1, ENSBTAP00000052439-D3, ENSBTAP00000001383-D1, ENSBTAP00000015277-D1, ENSP00000384262-D1, ENSP00000268668-D1, ENSP00000306397-D1, ENSP00000331897-D1, ENSP00000216962-D1, ENSP00000392709-D1, ENSP00000285093-D1, ENSP00000376894-D1, ENSP00000304592-D1, ENSBTAP00000007701-D1, ENSBTAP00000009923-D1, ENSP00000304006-D1, ENSP00000395337-D3, ENSBTAP00000007982-D1, ENSBTAP00000037577-D6, ENSBTAP00000017710-D1, ENSP00000345771-D1, ENSBTAP00000005730-D1, ENSP00000217426-D1, ENSP00000265132-D1, ENSP00000403265-D1, ENSP00000359991-D1, ENSBTAP00000040330-D1, ENSP00000362413-D2, ENSBTAP00000004079-D1, ENSBTAP00000006383-D1, ENSBTAP00000009440-D1, ENSBTAP00000015873-D1, ENSBTAP00000008907-D1, ENSBTAP00000027348-D1, ENSP00000229270-D1, ENSP00000378669-D1 |
| 30 | Complement and coagulation cascades | ENSBTAP00000006167-D1, ENSBTAP00000005713-D1, ENSP00000265023-D1, ENSBTAP00000018574-D1, ENSP00000384420-D1, ENSBTAP00000022979-D1, ENSBTAP00000023309-D1, ENSBTAP00000009798-D1, ENSBTAP00000009406-D1, ENSP00000306099-D1, ENSBTAP00000004927-D1, ENSBTAP00000042856-D1, ENSP00000306361-D1 |
| 31 | Bile secretion | ENSBTAP00000023552-D1, ENSBTAP00000013169-D1 |
| 32 | Phenylalanine, tyrosine and tryptophan biosynthesis | ENSBTAP00000009440-D1, ENSBTAP00000015873-D1 |
| 33 | Pathogenic Escherichia coli infection | ENSBTAP00000041398-D1, ENSBTAP00000015668-D1, ENSP00000355330-D1, ENSP00000346839-D1, ENSBTAP00000006923-D1, ENSP00000329165-D3, ENSP00000260356-D1, ENSBTAP00000009158-D3, ENSP00000363827-D1, ENSBTAP00000008132-D2, ENSP00000300527-D1, ENSP00000265132-D1, ENSBTAP00000047883-D15, ENSBTAP00000032779-D3, ENSBTAP00000001988-D1, ENSBTAP00000021529-D4 |
| 34 | Protein processing in endoplasmic reticulum | ENSBTAP00000017497-D1, ENSP00000324173-D1, ENSP00000368186-D4, ENSBTAP00000014883-D1, ENSP00000357076-D4, ENSP00000378294-D1, yakG038520, ENSP00000364805-D5, ENSBTAP00000002508-D1, ENSBTAP00000019970-D1, ENSP00000299767-D1, ENSBTAP00000040330-D1, ENSP00000300289-D1 |
| 35 | Proteoglycans in cancer | ENSBTAP00000024902-D1, ENSP00000384262-D1, ENSBTAP00000015668-D1, ENSP00000420213-D1, ENSP00000357076-D4, ENSP00000355330-D1, ENSP00000346839-D1, ENSBTAP00000006923-D1, ENSP00000260356-D1, ENSP00000363827-D1, ENSBTAP00000008132-D2, ENSP00000300527-D1, ENSP00000265132-D1, ENSBTAP00000009406-D1, ENSP00000306099-D1, ENSP00000358879-D1, ENSP00000327145-D1, ENSP00000306361-D1 |
| 36 | ECM-receptor interaction | ENSP00000343009-D1, ENSBTAP00000015668-D1, ENSP00000355330-D1, ENSP00000346839-D1, ENSBTAP00000006923-D1, ENSP00000260356-D1, ENSBTAP00000023750-D1, ENSP00000363827-D1, ENSP00000300527-D1, ENSP00000265132-D1, ENSBTAP00000009406-D1, ENSP00000306099-D1, ENSP00000306361-D1 |
| 37 | Toxoplasmosis | ENSP00000405382-D2, ENSBTAP00000017497-D1, ENSBTAP00000015668-D1, ENSP00000355330-D1, ENSP00000346839-D1, ENSBTAP00000006923-D1, ENSP00000260356-D1, ENSP00000364805-D5, ENSP00000363827-D1, ENSP00000300527-D1, ENSP00000265132-D1, ENSBTAP00000010351-D1 |
| 38 | MAPK signaling pathway | ENSBTAP00000017497-D1, ENSBTAP00000014883-D1, ENSP00000420213-D1, ENSP00000357076-D4, yakG038520, ENSP00000364805-D5, ENSP00000410452-D1, ENSP00000358879-D1, ENSP00000327145-D1 |
| 39 | Influenza A | ENSP00000405382-D2, ENSBTAP00000017497-D1, ENSP00000412582-D1, ENSP00000364805-D5, ENSP00000360671-D2, ENSBTAP00000008132-D2, ENSBTAP00000010351-D1 |
| 40 | Pertussis | ENSBTAP00000015668-D1, ENSP00000355330-D1, ENSP00000346839-D1, ENSBTAP00000006923-D1, ENSP00000260356-D1, ENSP00000384420-D1, ENSBTAP00000022979-D1, ENSBTAP00000020512-D4, ENSP00000363827-D1, ENSP00000300527-D1, ENSP00000265132-D1, ENSP00000306099-D1, ENSBTAP00000042856-D1, ENSP00000306361-D1 |
| 41 | Thyroid hormone synthesis | ENSP00000324173-D1, ENSBTAP00000013169-D1, ENSP00000299767-D1 |
| 42 | Small cell lung cancer | ENSP00000405382-D2, ENSBTAP00000015668-D1, ENSP00000355330-D1, ENSP00000346839-D1, ENSBTAP00000006923-D1, ENSP00000260356-D1, ENSP00000363827-D1, ENSP00000300527-D1, ENSP00000265132-D1 |
| 43 | Non-alcoholic fatty liver disease (NAFLD) | ENSP00000317159-D1, ENSP00000405382-D2, ENSP00000268668-D1, ENSP00000306397-D1, ENSP00000392709-D1, ENSBTAP00000014883-D1, ENSP00000357076-D4, yakG038520 |
| 44 | Bacterial invasion of epithelial cells | ENSBTAP00000024902-D1, ENSBTAP00000015668-D1, ENSP00000269122-D1, ENSP00000211998-D1, ENSP00000355330-D1, ENSP00000346839-D1, ENSBTAP00000006923-D1, ENSP00000260356-D1, ENSP00000363827-D1, ENSBTAP00000008132-D2, ENSP00000300527-D1, ENSP00000265132-D1 |
| 45 | Vitamin digestion and absorption | ENSBTAP00000026345-D1, ENSBTAP00000038799-D1 |
| 46 | Neuroactive ligand-receptor interaction | ENSP00000293190-D1, ENSBTAP00000009406-D1 |
| 47 | Proximal tubule bicarbonate reclamation | ENSBTAP00000009923-D1, ENSBTAP00000013169-D1 |
| 48 | Adrenergic signaling in cardiomyocytes | ENSBTAP00000019704-D1, ENSBTAP00000053653-D7, ENSBTAP00000013169-D1, ENSP00000350667-D1, ENSP00000350667-D3, ENSBTAP00000017182-D1 |
| 49 | Glyoxylate and dicarboxylate metabolism | ENSBTAP00000033696-D1, ENSBTAP00000027941-D1, ENSBTAP00000005730-D1 |
| 50 | Salivary secretion | ENSBTAP00000050833-D29, ENSBTAP00000016986-D1, ENSBTAP00000013169-D1 |
| 51 | Leishmaniasis | ENSBTAP00000015668-D1, ENSP00000355330-D1, ENSP00000346839-D1, ENSBTAP00000006923-D1, ENSP00000260356-D1, ENSBTAP00000022979-D1, ENSP00000363827-D1, ENSBTAP00000030521-D1, ENSP00000300527-D1, ENSP00000265132-D1, ENSP00000306099-D1, ENSBTAP00000010351-D1, ENSP00000306361-D1 |
| 52 | Phagosome | ENSBTAP00000041398-D1, ENSBTAP00000037042-D1, ENSBTAP00000015668-D1, ENSP00000265062-D1, ENSP00000355330-D1, ENSP00000346839-D1, ENSBTAP00000006923-D1, ENSP00000260356-D1, ENSBTAP00000009158-D3, ENSBTAP00000022979-D1, ENSBTAP00000023750-D1, ENSP00000363827-D1, ENSBTAP00000008132-D2, ENSBTAP00000030521-D1, ENSP00000300527-D1, ENSP00000265132-D1, ENSBTAP00000047883-D15, ENSBTAP00000009406-D1, ENSP00000306099-D1, ENSP00000306361-D1 |
| 53 | MicroRNAs in cancer | ENSP00000346839-D1, ENSBTAP00000006923-D1, ENSP00000260356-D1, ENSP00000350667-D1, ENSBTAP00000009406-D1, ENSP00000306099-D1, ENSP00000410452-D1, ENSP00000306361-D1 |
| 54 | PI3K-Akt signaling pathway | ENSP00000343009-D1, ENSP00000368186-D4, ENSBTAP00000015668-D1, ENSP00000355330-D1, ENSP00000346839-D1, ENSBTAP00000006923-D1, ENSP00000260356-D1, ENSP00000363827-D1, ENSBTAP00000030521-D1, ENSP00000300527-D1, ENSP00000299767-D1, ENSP00000265132-D1, ENSBTAP00000009406-D1, ENSP00000306099-D1, ENSBTAP00000032779-D3, ENSP00000306361-D1 |
| 55 | Salmonella infection | ENSP00000265062-D1, ENSP00000420213-D1, ENSBTAP00000008132-D2, ENSP00000358879-D1, ENSBTAP00000006465-D4, ENSP00000327145-D1, ENSP00000379613-D3 |
| 56 | Alzheimer's disease | ENSP00000317159-D1, ENSP00000405382-D2, ENSP00000293190-D1, ENSBTAP00000019704-D1, ENSP00000268668-D1, ENSP00000306397-D1, ENSP00000392709-D1, ENSBTAP00000037577-D6, ENSBTAP00000017710-D1, ENSBTAP00000013354-D1 |
| 57 | cAMP signaling pathway | ENSP00000293190-D1, ENSBTAP00000019704-D1, ENSP00000357076-D4, ENSBTAP00000013169-D1, ENSBTAP00000017182-D1, ENSBTAP00000015248-D2 |
| 58 | Shigellosis | ENSBTAP00000015668-D1, ENSP00000211998-D1, ENSP00000355330-D1, ENSP00000346839-D1, ENSBTAP00000006923-D1, ENSP00000260356-D1, ENSP00000363827-D1, ENSBTAP00000008132-D2, ENSP00000300527-D1, ENSP00000265132-D1, ENSBTAP00000006465-D4 |
| 59 | Fatty acid biosynthesis | ENSP00000304592-D1 |
| 60 | Protein export | ENSP00000324173-D1 |
| 61 | Nicotine addiction | ENSP00000293190-D1 |
| 62 | D-Glutamine and D-glutamate metabolism | ENSBTAP00000009923-D1 |
| 63 | ABC transporters | ENSBTAP00000023552-D1 |
| 64 | Nitrogen metabolism | ENSBTAP00000009923-D1 |
| 65 | Pyruvate metabolism | ENSBTAP00000033696-D1, ENSBTAP00000052439-D3, ENSP00000395337-D3, ENSP00000403265-D1 |
| 66 | Viral myocarditis | ENSP00000405382-D2, ENSBTAP00000037042-D1, ENSBTAP00000053653-D7, ENSP00000346839-D1, ENSBTAP00000022979-D1, ENSP00000363827-D1, ENSBTAP00000008132-D2, ENSBTAP00000030521-D1, ENSP00000306099-D1, ENSP00000306361-D1 |
| 67 | Platelet activation | ENSBTAP00000015668-D1, ENSP00000355330-D1, ENSP00000346839-D1, ENSBTAP00000006923-D1, ENSP00000260356-D1, ENSP00000363827-D1, ENSBTAP00000008132-D2, ENSP00000300527-D1, ENSP00000265132-D1, ENSBTAP00000009406-D1, ENSP00000306099-D1, ENSP00000306361-D1 |
| 68 | Peroxisome | ENSBTAP00000027941-D1, ENSP00000331897-D1, ENSBTAP00000027348-D1 |
| 69 | Huntington's disease | ENSP00000317159-D1, ENSP00000405382-D2, ENSP00000268668-D1, ENSP00000269122-D1, ENSP00000306397-D1, ENSP00000392709-D1, ENSP00000355330-D1, ENSBTAP00000017710-D1, ENSP00000360671-D2 |
| 70 | Fatty acid metabolism (no map in kegg database) | ENSP00000285093-D1, ENSP00000304592-D1 |
| 71 | cGMP-PKG signaling pathway | ENSBTAP00000019704-D1, ENSBTAP00000053653-D7, ENSBTAP00000013169-D1, ENSP00000360671-D2, ENSBTAP00000017182-D1, ENSBTAP00000015248-D2 |
| 72 | Herpes simplex infection | ENSP00000405382-D2, ENSBTAP00000037042-D1, ENSBTAP00000014883-D1, ENSBTAP00000022979-D1, ENSBTAP00000010351-D1 |
| 73 | Hepatitis B | ENSP00000405382-D2, ENSP00000363827-D1, ENSBTAP00000032779-D3, ENSBTAP00000010351-D1 |
| 74 | Cysteine and methionine metabolism | ENSP00000395337-D3, ENSP00000217426-D1, ENSBTAP00000009440-D1, ENSBTAP00000015873-D1 |
| 75 | Protein digestion and absorption | ENSBTAP00000015668-D1, ENSBTAP00000013169-D1, ENSP00000300527-D1, ENSP00000265132-D1 |
| 76 | Antigen processing and presentation | ENSBTAP00000017497-D1, ENSBTAP00000037042-D1, ENSP00000324173-D1, ENSP00000368186-D4, ENSP00000364805-D5, ENSP00000300289-D1 |
| 77 | Propanoate metabolism | ENSBTAP00000033696-D1, ENSP00000395337-D3, ENSBTAP00000007982-D1 |
| 78 | Insulin resistance | ENSBTAP00000015277-D1, ENSP00000216962-D1, ENSBTAP00000023750-D1 |
| 79 | Fructose and mannose metabolism | ENSP00000345771-D1, ENSP00000229270-D1, ENSP00000378669-D1 |
| 80 | AMPK signaling pathway | ENSP00000304592-D1, ENSBTAP00000005581-D1, ENSP00000345771-D1, ENSBTAP00000023750-D1, ENSBTAP00000044443-D1 |
| 81 | Parkinson's disease | ENSP00000317159-D1, ENSP00000405382-D2, ENSP00000268668-D1, ENSP00000306397-D1, ENSP00000392709-D1, ENSBTAP00000017710-D1, ENSP00000360671-D2 |
| 82 | Rap1 signaling pathway | ENSBTAP00000015668-D1, ENSP00000357076-D4, ENSP00000355330-D1, ENSP00000346839-D1, ENSBTAP00000006923-D1, ENSP00000260356-D1, ENSBTAP00000022979-D1, ENSP00000363827-D1, ENSBTAP00000008132-D2, ENSP00000300527-D1, ENSP00000265132-D1, ENSBTAP00000009406-D1, ENSP00000306099-D1, ENSBTAP00000006465-D4, ENSP00000306361-D1 |
| 83 | Staphylococcus aureus infection | ENSP00000329165-D3, ENSBTAP00000022979-D1, ENSBTAP00000030521-D1, ENSBTAP00000009798-D1, ENSP00000306099-D1, ENSBTAP00000001988-D1, ENSBTAP00000042856-D1, ENSP00000306361-D1 |
| 84 | Endocrine and other factor-regulated calcium reabsorption | ENSP00000269122-D1, ENSBTAP00000013169-D1 |
| 85 | NOD-like receptor signaling pathway | ENSP00000368186-D4, ENSP00000299767-D1 |
| 86 | Tryptophan metabolism | ENSP00000414662-D1, ENSBTAP00000027941-D1 |
| 87 | Prolactin signaling pathway | ENSBTAP00000003409-D1, ENSBTAP00000010351-D1 |
| 88 | Oxytocin signaling pathway | ENSBTAP00000005581-D1, ENSBTAP00000014306-D5, ENSBTAP00000008132-D2, ENSBTAP00000015248-D2 |
| 89 | Measles | ENSBTAP00000017497-D1, ENSP00000364805-D5, ENSBTAP00000030521-D1, ENSBTAP00000010351-D1 |
| 90 | Estrogen signaling pathway | ENSBTAP00000017497-D1, ENSP00000368186-D4, ENSP00000364805-D5, ENSP00000299767-D1 |
| 91 | Spliceosome | ENSBTAP00000022514-D25, ENSBTAP00000017497-D1, ENSP00000412582-D1, yakG032579, ENSP00000364805-D5, ENSP00000338095-D1, ENSBTAP00000022514-D11 |
| 92 | Tight junction | ENSBTAP00000053653-D7, ENSBTAP00000020395-D1, ENSP00000284770-D1, ENSBTAP00000008132-D2, ENSBTAP00000015248-D2, ENSBTAP00000049867-D3, ENSP00000379613-D3 |
| 93 | Cell adhesion molecules (CAMs) | ENSBTAP00000037042-D1, ENSBTAP00000015668-D1, ENSP00000355330-D1, ENSP00000346839-D1, ENSBTAP00000006923-D1, ENSP00000260356-D1, ENSBTAP00000022979-D1, ENSP00000363827-D1, ENSP00000300527-D1, ENSP00000265132-D1, ENSP00000306099-D1, ENSP00000395463-D14, ENSP00000306361-D1 |
| 94 | Hematopoietic cell lineage | ENSBTAP00000037042-D1, ENSP00000346839-D1, ENSBTAP00000006923-D1, ENSBTAP00000023750-D1, ENSBTAP00000030521-D1, ENSBTAP00000009406-D1, ENSP00000306099-D1, ENSP00000306361-D1 |
| 95 | Gap junction | ENSBTAP00000041398-D1, ENSBTAP00000009158-D3, ENSBTAP00000047883-D15 |
| 96 | Glutathione metabolism | ENSP00000331897-D1, ENSP00000335620-D3, ENSBTAP00000027348-D1 |
| 97 | Arginine and proline metabolism | ENSBTAP00000004079-D1, ENSBTAP00000009440-D1, ENSBTAP00000015873-D1 |
| 98 | Inositol phosphate metabolism | ENSP00000229270-D1 |
| 99 | Carbohydrate digestion and absorption | ENSBTAP00000013169-D1 |
| 100 | Fatty acid elongation | ENSP00000285093-D1 |
| 101 | Inflammatory bowel disease (IBD) | ENSBTAP00000010351-D1 |
| 102 | Vascular smooth muscle contraction | ENSBTAP00000014306-D5, ENSP00000224784-D2, ENSBTAP00000015248-D2, ENSP00000379613-D3 |
| 103 | Pathways in cancer | ENSP00000405382-D2, ENSP00000368186-D4, ENSBTAP00000015668-D1, ENSP00000355330-D1, ENSP00000335620-D3, ENSP00000346839-D1, ENSBTAP00000006923-D1, ENSP00000260356-D1, ENSP00000363827-D1, ENSP00000300527-D1, ENSP00000299767-D1, ENSP00000265132-D1, ENSBTAP00000010351-D1 |
| 104 | Calcium signaling pathway | ENSP00000293190-D1, ENSBTAP00000019704-D1, ENSP00000360671-D2, ENSBTAP00000030521-D1, ENSBTAP00000017182-D1 |
| 105 | Oxidative phosphorylation | ENSP00000317159-D1, ENSP00000268668-D1, ENSP00000306397-D1, ENSP00000392709-D1, ENSBTAP00000017710-D1 |
| 106 | Hippo signaling pathway | ENSBTAP00000022979-D1, ENSBTAP00000008132-D2, ENSP00000306099-D1, ENSBTAP00000032779-D3, ENSP00000306361-D1 |
| 107 | Gastric acid secretion | ENSBTAP00000013169-D1, ENSBTAP00000008132-D2 |
| 108 | Hippo signaling pathway - fly | ENSBTAP00000008132-D2, ENSBTAP00000032779-D3 |
| 109 | Starch and sucrose metabolism | ENSBTAP00000015277-D1, ENSP00000216962-D1 |
| 110 | Pancreatic secretion | ENSBTAP00000019704-D1, ENSBTAP00000013169-D1 |
| 111 | TNF signaling pathway | ENSBTAP00000014883-D1, ENSP00000357076-D4, yakG038520 |
| 112 | Viral carcinogenesis | ENSBTAP00000037042-D1, ENSBTAP00000013453-D5, ENSBTAP00000024902-D1, ENSP00000375736-D1, ENSBTAP00000022979-D1, ENSP00000403265-D1, ENSBTAP00000049867-D3, ENSBTAP00000032779-D3 |
| 113 | PPAR signaling pathway | ENSP00000284770-D1, ENSBTAP00000053003-D4, ENSBTAP00000022375-D1, ENSBTAP00000023750-D1 |
| 114 | Neurotrophin signaling pathway | ENSBTAP00000014883-D1, ENSP00000357076-D4, yakG038520, ENSBTAP00000017863-D1 |
| 115 | Axon guidance | ENSBTAP00000015668-D1, ENSP00000355330-D1, ENSP00000346839-D1, ENSBTAP00000006923-D1, ENSP00000260356-D1, ENSBTAP00000020512-D4, ENSP00000363827-D1, ENSP00000300527-D1, ENSP00000265132-D1 |
| 116 | Toll-like receptor signaling pathway | ENSBTAP00000006923-D1, ENSBTAP00000010351-D1 |
| 117 | Valine, leucine and isoleucine degradation | ENSBTAP00000033696-D1, ENSP00000285093-D1 |
| 118 | Chemokine signaling pathway | ENSBTAP00000024902-D1, ENSP00000357076-D4, ENSBTAP00000010351-D1 |
| 119 | Prostate cancer | ENSP00000368186-D4, ENSP00000335620-D3, ENSP00000299767-D1 |
| 120 | Glycosaminoglycan degradation | ENSP00000384262-D1 |
| 121 | Cocaine addiction | ENSP00000293190-D1 |
| 122 | Sulfur metabolism | ENSP00000405382-D2 |
| 123 | Drug metabolism - cytochrome P450 | ENSP00000335620-D3 |
| 124 | Ether lipid metabolism | ENSP00000304006-D1 |
| 125 | Adipocytokine signaling pathway | ENSBTAP00000023750-D1 |
| 126 | Phototransduction - fly | ENSBTAP00000008132-D2 |
| 127 | Renin-angiotensin system | ENSP00000367697-D1 |
| 128 | Insulin signaling pathway | ENSBTAP00000015277-D1, ENSP00000216962-D1, ENSP00000304592-D1, ENSP00000284770-D1 |
| 129 | Natural killer cell mediated cytotoxicity | ENSBTAP00000037042-D1, ENSP00000357076-D4, ENSBTAP00000022979-D1, ENSBTAP00000030521-D1, ENSP00000306099-D1, ENSP00000306361-D1 |
| 130 | B cell receptor signaling pathway | ENSP00000357076-D4, ENSBTAP00000030521-D1, ENSBTAP00000012837-D1 |
| 131 | Purine metabolism | ENSP00000376894-D1, ENSP00000403265-D1 |
| 132 | Pentose phosphate pathway | ENSP00000345771-D1, ENSP00000378669-D1 |
| 133 | Adherens junction | ENSP00000211998-D1, ENSBTAP00000014883-D1, ENSP00000284770-D1, yakG038520, ENSBTAP00000008132-D2, ENSBTAP00000049867-D3 |
| 134 | Amoebiasis | ENSP00000211998-D1, ENSP00000265062-D1, ENSP00000346839-D1, ENSBTAP00000022979-D1, ENSBTAP00000030521-D1, ENSBTAP00000049867-D3, ENSP00000306099-D1, ENSP00000306361-D1 |
| 135 | N-Glycan biosynthesis | ENSBTAP00000040330-D1 |
| 136 | Chemical carcinogenesis | ENSP00000335620-D3 |
| 137 | Metabolism of xenobiotics by cytochrome P450 | ENSP00000335620-D3 |
| 138 | Vibrio cholerae infection | ENSBTAP00000008132-D2 |
| 139 | Aldosterone-regulated sodium reabsorption | ENSBTAP00000013169-D1 |
| 140 | Lysine degradation | ENSP00000414662-D1 |
| 141 | Amphetamine addiction | ENSP00000293190-D1 |
| 142 | Fatty acid degradation | ENSP00000285093-D1 |
| 143 | Jak-STAT signaling pathway | ENSBTAP00000010351-D1 |
| 144 | Bladder cancer | ENSP00000260356-D1 |
| 145 | Pyrimidine metabolism | ENSP00000376894-D1 |
| 146 | Cell cycle | ENSBTAP00000032779-D3 |
| 147 | African trypanosomiasis | ENSBTAP00000050256-D2, ENSBTAP00000051337-D10, ENSBTAP00000030521-D1 |
| 148 | Galactose metabolism | ENSBTAP00000007701-D1, ENSP00000345771-D1 |
| 149 | Primary immunodeficiency | ENSBTAP00000037042-D1, ENSP00000374847-D1, ENSBTAP00000030521-D1, ENSBTAP00000048809-D1 |
| 150 | Rheumatoid arthritis | ENSBTAP00000022979-D1, ENSBTAP00000030521-D1, ENSP00000306099-D1, ENSP00000306361-D1 |
| 151 | Sphingolipid signaling pathway | ENSBTAP00000014883-D1, ENSP00000357076-D4, yakG038520 |
| 152 | Hepatitis C | ENSBTAP00000010351-D1 |
| 153 | Synaptic vesicle cycle | ENSP00000269122-D1 |
| 154 | Insulin secretion | ENSBTAP00000013169-D1 |
| 155 | Progesterone-mediated oocyte maturation | ENSP00000368186-D4 |
| 156 | Apoptosis | ENSP00000405382-D2 |
| 157 | T cell receptor signaling pathway | ENSBTAP00000037042-D1, ENSP00000357076-D4 |
| 158 | Systemic lupus erythematosus | ENSBTAP00000013453-D5, ENSP00000375736-D1, ENSBTAP00000022979-D1, ENSBTAP00000030521-D1, ENSBTAP00000049867-D3, ENSBTAP00000042856-D1 |
| 159 | Alcoholism | ENSP00000293190-D1, ENSBTAP00000013453-D5, ENSP00000375736-D1 |
| 160 | HTLV-I infection | ENSBTAP00000037042-D1, ENSBTAP00000022979-D1, ENSP00000360671-D2, ENSP00000306099-D1, ENSP00000306361-D1 |
| 161 | Tuberculosis | ENSP00000405382-D2, ENSP00000265062-D1, ENSBTAP00000022979-D1, ENSBTAP00000030521-D1, ENSP00000306099-D1, ENSBTAP00000010351-D1, ENSP00000306361-D1 |
| 162 | Intestinal immune network for IgA production | ENSP00000346839-D1, ENSBTAP00000030521-D1 |
| 163 | mRNA surveillance pathway | ENSP00000412582-D1 |
| 164 | FoxO signaling pathway | ENSBTAP00000027941-D1 |
| 165 | Graft-versus-host disease | ENSBTAP00000037042-D1 |
| 166 | Type II diabetes mellitus | ENSP00000403265-D1 |
| 167 | Type I diabetes mellitus | ENSBTAP00000037042-D1 |
| 168 | Glutamatergic synapse | ENSP00000293190-D1 |
| 169 | Long-term potentiation | ENSP00000293190-D1 |
| 170 | TGF-beta signaling pathway | ENSP00000260356-D1 |
| 171 | Pancreatic cancer | ENSBTAP00000010351-D1 |
| 172 | Ribosome | ENSBTAP00000037200-D1, ENSBTAP00000038763-D5, ENSBTAP00000003962-D1 |
| 173 | Epstein-Barr virus infection | ENSBTAP00000017497-D1, ENSBTAP00000037042-D1, ENSP00000346839-D1, ENSP00000364805-D5, ENSBTAP00000030521-D1, ENSBTAP00000032779-D3 |
| 174 | Autoimmune thyroid disease | ENSBTAP00000037042-D1, ENSBTAP00000030521-D1 |
| 175 | Allograft rejection | ENSBTAP00000037042-D1, ENSBTAP00000030521-D1 |
| 176 | Transcriptional misregulation in cancer | ENSBTAP00000013453-D5, ENSP00000346839-D1, ENSBTAP00000030521-D1 |
| 177 | beta-Alanine metabolism | ENSBTAP00000001383-D1 |
| 178 | Choline metabolism in cancer | ENSP00000284770-D1 |
| 179 | Arachidonic acid metabolism | ENSP00000265132-D1 |
| 180 | RNA degradation | ENSP00000345771-D1 |
| 181 | Prion diseases | ENSBTAP00000017497-D1, ENSP00000324173-D1, ENSP00000364805-D5 |
| 182 | Fc epsilon RI signaling pathway | ENSP00000357076-D4, ENSBTAP00000030521-D1 |
| 183 | Circadian entrainment | ENSP00000293190-D1 |
| 184 | Chagas disease (American trypanosomiasis) | ENSBTAP00000022979-D1 |
| 185 | Colorectal cancer | ENSP00000405382-D2 |
| 186 | Vasopressin-regulated water reabsorption | ENSBTAP00000017863-D1 |
| 187 | Oocyte meiosis | ENSBTAP00000032779-D3 |
| 188 | Endocytosis | ENSBTAP00000017497-D1, ENSBTAP00000037042-D1, ENSP00000269122-D1, ENSP00000265062-D1, ENSBTAP00000002174-D3, ENSP00000364805-D5 |
| 189 | Fc gamma R-mediated phagocytosis | ENSP00000357076-D4, ENSBTAP00000020512-D4, ENSBTAP00000030521-D1 |
| 190 | NF-kappa B signaling pathway | ENSBTAP00000030521-D1 |
| 191 | Asthma | ENSBTAP00000030521-D1 |
| 192 | VEGF signaling pathway | ENSBTAP00000024902-D1 |
| 193 | RNA transport | ENSP00000412582-D1, ENSP00000339053-D13 |
| 194 | Proteasome | ENSP00000271308-D1 |
| 195 | Lysosome | ENSP00000269122-D1, ENSP00000388762-D1, ENSBTAP00000003073-D1 |
| 196 | Phospholipase D signaling pathway | ENSBTAP00000030521-D1 |
